# Evaluation of Plasmodium vivax Cell-Traversal Protein for Ookinetes and Sporozoites as a Preerythrocytic P. vivax Vaccine

**DOI:** 10.1128/CVI.00501-16

**Published:** 2017-04-05

**Authors:** Eduardo Alves, Ahmed M. Salman, Fabiana Leoratti, Cesar Lopez-Camacho, Martha Eva Viveros-Sandoval, Amar Lall, Aadil El-Turabi, Martin F. Bachmann, Adrian V. S. Hill, Chris J. Janse, Shahid M. Khan, Arturo Reyes-Sandoval

**Affiliations:** a The Jenner Institute, Nuffield Department of Medicine, University of Oxford, Oxford, United Kingdom; b Leiden Malaria Research Group, Department of Parasitology, Center of Infectious Diseases, Leiden University Medical Center, Leiden, The Netherlands; c Laboratorio de Hemostasia y Biología Vascular, División de Estudios de Posgrado, Facultad de Ciencias Médicas y Biológicas Dr. Ignacio Chávez, Universidad Michoacana de San Nicolás de Hidalgo, Morelia, Michoacán, Mexico; d Immunology, RIA, Inselspital, University of Bern, Bern, Switzerland; CDC

**Keywords:** CelTOS, malaria, Plasmodium, preerythrocytic, vaccine, vivax

## Abstract

Four different vaccine platforms, each targeting the human malaria parasite Plasmodium vivax cell-traversal protein for ookinetes and sporozoites (*Pv*CelTOS), were generated and assessed for protective efficacy. These platforms consisted of a recombinant chimpanzee adenoviral vector 63 (ChAd63) expressing *Pv*CelTOS (Ad), a recombinant modified vaccinia virus Ankara expressing *Pv*CelTOS (MVA), *Pv*CelTOS conjugated to bacteriophage Qβ virus-like particles (VLPs), and a recombinant *Pv*CelTOS protein expressed in eukaryotic HEK293T cells (protein). Inbred BALB/c mice and outbred CD-1 mice were immunized using the following prime-boost regimens: Ad-MVA, Ad-VLPs, and Ad-protein. Protective efficacy against sporozoite challenge was assessed after immunization using a novel chimeric rodent Plasmodium berghei parasite (*Pb-Pv*CelTOS). This chimeric parasite expresses P. vivax CelTOS in place of the endogenous P. berghei CelTOS and produces fully infectious sporozoites. A single Ad immunization in BALB/c and CD-1 mice induced anti-*Pv*CelTOS antibodies which were boosted efficiently using MVA, VLP, or protein immunization. *Pv*CelTOS-specific gamma interferon- and tumor necrosis factor alpha-producing CD8^+^ T cells were induced at high frequencies by all prime-boost regimens in BALB/c mice but not in CD-1 mice; in CD-1 mice, they were only marginally increased after boosting with MVA. Despite the induction of anti-*Pv*CelTOS antibodies and *Pv*CelTOS-specific CD8^+^ T-cell responses, only low levels of protective efficacy against challenge with *Pb-Pv*CelTOS sporozoites were obtained using any immunization strategy. In BALB/c mice, no immunization regimens provided significant protection against a *Pb-Pv*CelTOS chimeric sporozoite challenge. In CD-1 mice, modest protective efficacy against challenge with chimeric P. berghei sporozoites expressing either *Pv*CelTOS or P. falciparum CelTOS was observed using the Ad-protein vaccination regimen.

## INTRODUCTION

Plasmodium vivax is the most widely distributed human malaria parasite in the world. It is a major health risk to 2.85 billion people and is considered the most difficult species of malaria parasite to control and eliminate from regions of endemicity (1). This is largely due to the parasite's ability to remain latent in the liver of infected people, reactivating weeks or even years after an initial infection (2, 3). As with Plasmodium falciparum, no effective vaccine offering protection against P. vivax infection has yet been licensed. The preerythrocytic stage (sporozoite and infected liver stage) continues to be the most attractive target for vaccine development (4). The most advanced vaccine against P. falciparum is RTS,S/AS01, which aims to prevent infection by stimulating immune responses against the major Plasmodium sporozoite surface antigen circumsporozoite protein (CSP). A phase III trial of RTS,S/AS01 conducted at 11 sites in seven African countries demonstrated 28% efficacy for 5- to 17-month-old children and 18% efficacy for 6- to 12-week-old infants with three doses over the entire course of the study (∼3 to 4 years of follow-up) (5).

Despite the difficulty in testing P. vivax vaccine candidates in controlled human infection studies, P. vivax CSP (*Pv*CSP) is also being actively investigated as a preerythrocytic stage vaccine (6–9). Various vaccine platforms and strategies have been tested, and protective efficacy against infection has been demonstrated in nonhuman primates (10) and in immunized mice using chimeric rodent malaria parasites expressing *Pv*CSP (11, 12).

While the protective efficacy of RTS,S/AS01 is encouraging, improvements will be necessary to induce higher levels of protective immunity as well as broad strain-transcending immunity. A strategy for increasing the effectiveness of subunit malaria vaccines is the use of formulations that incorporate multiple parasite antigens targeting several stages of the parasite cycle (13). A number of studies have sought to identify alternative sporozoite antigens to CSP that similarly induce protective immunity. One candidate antigen is the cell-traversal protein for ookinetes and sporozoites (CelTOS), a secretory microneme protein that is required for parasite traversal of host cells both for ookinetes in the mosquito and for sporozoites (14). In studies using peripheral blood mononuclear cells (PBMCs) from volunteers immunized with irradiated P. falciparum sporozoites, the *ex vivo* gamma interferon (IFN-γ) enzyme-linked immunosorbent spot assay responses induced by P. falciparum CelTOS (*Pf*CelTOS) peptides correlated the best with protection in volunteers (15). Anti-*Pf*CelTOS antibodies have been shown to inhibit sporozoite motility and invasion of hepatocytes *in vitro*, and immunization of mice with recombinant *Pf*CelTOS has induced sterile protection against a heterologous challenge with P. berghei sporozoites (16, 17). Immunization of mice with live-attenuated Shigella expressing the rodent Plasmodium berghei CelTOS (*Pb*CelTOS) has been shown to induce protective efficacy against P. berghei sporozoite challenge (18). In addition, a DNA vaccine coding for *Pf*CelTOS has been shown to induce humoral and cellular responses against the protein in mice and nonhuman primates (19).

In contrast to *Pf*CelTOS, studies investigating P. vivax CelTOS (*Pv*CelTOS) as a potential preerythrocytic stage vaccine candidate have not yet been reported. Our interest in this candidate came from reports of its good immunogenicity and protective efficacy against infection in rodent models as well as from the conserved nature of *Pv*CelTOS, with some evidence of cross-species protection being provided (17, 20). In this study, we report on the generation and analysis of four different clinically relevant vaccine platforms to target *Pv*CelTOS. These platforms are based on previous studies targeting various vaccine candidate antigens in both P. falciparum and P. vivax. The recombinant chimpanzee adenoviral (Ad) vector 63 (ChAd63) and modified vaccinia virus Ankara (MVA) vectors have proven effective in generating protective immunity against a variety of antigens in animal models and in humans (21). An initial prime immunization with ChAd63 expressing a Plasmodium antigen followed by a boost immunization with MVA expressing the same parasite antigen has been shown to elicit exceptionally high antigen-specific T-cell responses (22). Virus-like particles (VLPs) are self-assembly systems that spontaneously form virus-shaped particles following expression of one or more viral proteins (23). RTS,S, for example, is a VLP based on the hepatitis B surface antigen (24). VLPs are able to induce strong B-cell responses in the absence of adjuvants by efficiently cross-linking specific receptors on B cells (25). In this study, we used VLPs derived from the bacteriophage Qβ which spontaneously assemble around bacterial RNA following expression in Escherichia coli (26). Qβ VLPs have been shown to be immunogenic in clinical studies (27). In addition, we expressed *Pv*CelTOS as a protein using HEK293T cells, a eukaryotic cell expression system. This protein was coadministered with Matrix-M, a saponin-based adjuvant that is mixed with synthetic cholesterol and a phospholipid and that is thus able to induce both cellular and humoral immune responses (28). In this study, we evaluated the *Pv*CelTOS-specific humoral and cellular immune responses elicited by four different immunization strategies using these platforms. In addition, we analyzed the protective efficacy conferred by these four vaccination protocols in mice using a novel challenge model employing a chimeric rodent Plasmodium (P. berghei) parasite (*Pb-Pv*CelTOS). This parasite expresses P. vivax CelTOS in place of the endogenous P. berghei CelTOS. Sporozoites of these chimeric parasites were used to challenge mice that were previously immunized with the various vaccine platforms. Moreover, to address the potential cross-species efficacy afforded by a P. vivax CelTOS vaccine candidate, we made use of a wild-type P. berghei parasite and a chimeric parasite expressing P. falciparum CelTOS recently described in the literature (29, 30).

## RESULTS

### Vaccine platforms targeting *Pv*CelTOS.

**FIG 1:**
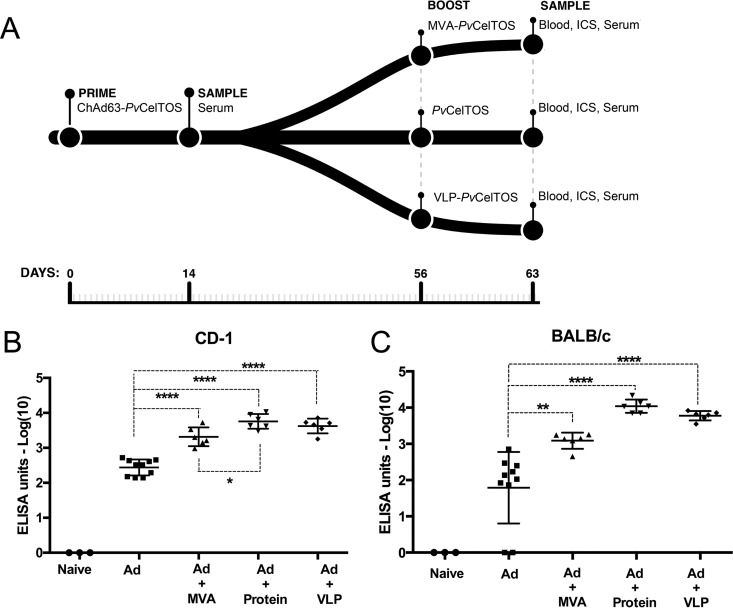
Vaccination regimens and induction of antibody responses against P. vivax CelTOS in outbred CD-1 and inbred BALB/c mice. (A) Flowchart of the vaccination regimens used in this study. Three groups of 6 mice each were primed with the viral ChAd63 vector (Ad) expressing *Pv*CelTOS (ChAd63-*Pv*CelTOS). These groups were subsequently boosted with (i) the MVA viral vector expressing *Pv*CelTOS (MVA-*Pv*CelTOS), (ii) the *Pv*CelTOS protein expressed in eukaryotic HEK293T cells (*Pv*CelTOS), or (iii) the *Pv*CelTOS protein conjugated to bacteriophage Qβ VLPs (VLP-*Pv*CelTOS). Blood samples were collected at 14 days after the Ad prime and at day 63 after the boost. (B) Endpoint titer ELISA showing the total IgG antibody response against recombinant *Pv*CelTOS protein in CD-1 mice after priming with Ad (day 14) or after boosting with MVA, protein, or VLPs (day 63), as shown in panel A. Means with standard errors of the means (SEMs) are shown. *P* values were determined by Tukey's multiple-comparison test. *, *P* < 0.05; ****, *P* < 0.0001. (C) Endpoint titer ELISA showing the total IgG antibody response against recombinant *Pv*CelTOS protein in BALB/c mice after priming with Ad (day 14) or after boosting with MVA, protein, or VLPs (day 63), as shown in panel A. Means with SEMs are shown. *P* values were determined by Tukey's multiple-comparison test. **, *P* < 0.01; ****, *P* < 0.0001.

We developed four vaccine platforms to induce immune responses directed against *Pv*CelTOS: a recombinant chimpanzee adenoviral vector (ChAd63) expressing *Pv*CelTOS (Ad), a recombinant MVA vector expressing *Pv*CelTOS (MVA), *Pv*CelTOS conjugated to bacteriophage Qβ virus-like particles (VLPs), and the *Pv*CelTOS protein produced in eukaryotic HEK293T cells (protein). VLPs and protein were delivered using the Matrix-M adjuvant. To prime immune responses in BALB/c and CD-1 mice, ChAd63-*Pv*CelTOS was injected intramuscularly. The other three vaccine platforms were injected intramuscularly 8 weeks later to boost responses, such that three groups of mice (*n* = 6 each) were immunized with the following: Ad-MVA, Ad-protein, and Ad-VLPs (Fig. 1A). Serum and peripheral blood mononuclear cells (PBMCs) were collected 7 days after priming and after boosting to assess the humoral and cellular immune responses. We followed this prime-boost approach using a chimpanzee adenovirus followed by other platforms, as it has previously been described that an initial adenovirus prime can benefit subsequent boosting immunizations, as well as support the induction of T effector memory (Tem) cells that correlate with protection upon a sporozoite challenge (31, 32).

### Anti-*Pv*CelTOS antibodies induced by prime-boost vaccination regimens.

CD-1 mice primed with Ad produced a mean anti-*Pv*CelTOS antibody titer (log_10_) of 2.44 ± 0.229 (standard deviation [SD]) 1 week after priming. Antibody levels increased significantly after boosting with all three vaccine platforms (MVA, VLPs, and protein) (*P* < 0.001) (Fig. 1B). The MVA boost resulted in a mean titer of 3.32 ± 0.269 (SD), the protein boost resulted in a mean titer of 3.76 ± 0.211 (SD), and the VLP boost resulted in a mean titer of 3.63 ± 0.209 (SD). Immunization of BALB/c mice produced similar antibody responses, with a mean titer of 1.79 ± 0.987 (SD) following Ad priming and mean titers after boost of 3.09 ± 0.222 (SD) following immunization with Ad-MVA, 4.04 ± 0.185 (SD) with Ad-protein, and 3.78 ± 0.13 (SD) with Ad-VLPs (Fig. 1C). The titers were significantly higher after immunization with MVA (*P* < 0.01), protein (*P* < 0.001), and VLPs (*P* < 0.001) than after Ad priming. Thus, antibody responses were boosted with all three vaccine platforms, and boosting with protein in the Matrix-M adjuvant consistently elicited the highest titers. Although no significant differences in titers were observed between the platforms upon a boost in BALB/c mice, the titer obtained with Ad-protein was significantly higher than that obtained with Ad-MVA in CD-1 mice.

### Anti-*Pv*CelTOS T-cell responses induced by prime-boost vaccination regimens.

**FIG 2:**
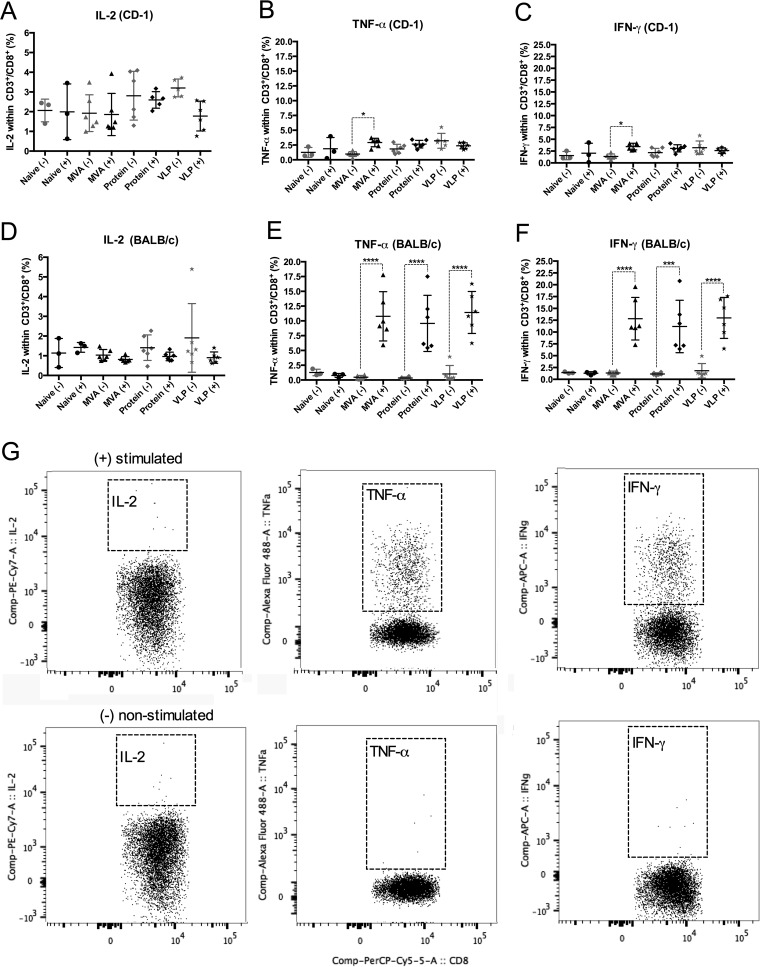
(A to F) *Ex vivo* production of IL-2, TNF-α, and IFN-γ by CD3^+^/CD8^+^ cells upon *Pv*CelTOS stimulation of PBMCs obtained from immunized CD-1 and BALB/c mice. Samples were collected 1 week after boosting with MVA, protein, and VLPs, as shown in Fig. 1A, and PBMCs were analyzed with (+) or without (−) stimulation with a peptide pool of *Pv*CelTOS (*n* = 3 for naive mice, *n* = 6 for the other groups). (A to C) Frequencies of CD3^+^/CD8^+^ cells in CD-1 mice producing IL-2 (A), TNF-α (B), and IFN-γ (C); (D to F) frequencies of CD3^+^/CD8^+^ cells in BALB/c mice producing IL-2 (D), TNF-α (E), and IFN-γ (F). MVA, group boosted with MVA-*Pv*CelTOS; Protein, group boosted with *Pv*CelTOS protein plus the Matrix-M adjuvant; VLP, group boosted with *Pv*CelTOS coupled to VLPs plus the Matrix-M adjuvant. Means with SEMs are represented. *P* values were determined by one-way ANOVA followed by Tukey's multiple-comparison test. *, *P* < 0.05; ***, *P* < 0.001; ****, *P* < 0.0001. (G) Representative dot plots showing the production of the three cytokines in the gated CD3^+^/CD8^+^ population obtained from one representative BALB/c mouse vaccinated with a ChAd63 prime and a boost with MVA expressing *Pv*CelTOS. PerCP, peridinin chlorophyll protein; Comp, compensated; A, area.

**FIG 3:**
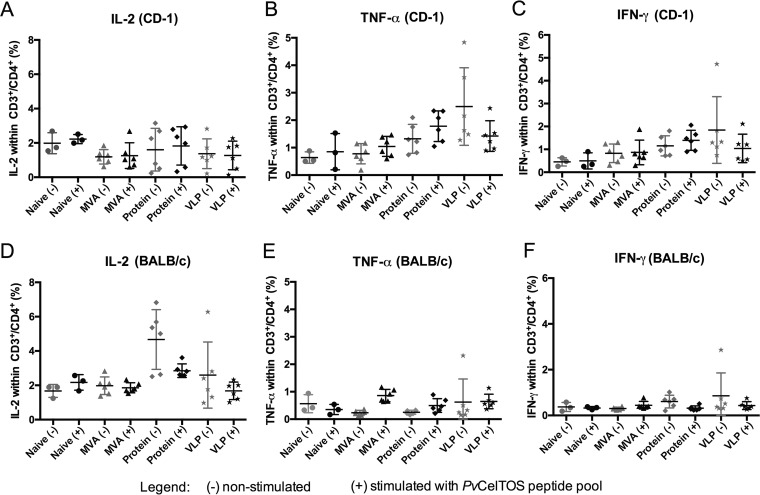
*Ex vivo* production of IL-2, TNF-α, and IFN-γ by CD3^+^/CD4^+^ cells upon *Pv*CelTOS stimulation of PBMCs obtained from vaccinated CD-1 and BALB/c mice. Samples were collected 1 week after boosting with MVA, protein, and VLPs, as shown in Fig. 1A, and PBMCs were analyzed with (+) or without (−) stimulation with a peptide pool of *Pv*CelTOS (*n* = 3 for naive mice, *n* = 6 for the other groups). (A to C) Frequencies of CD3^+^/CD4^+^ cells in CD-1 mice producing IL-2 (A), TNF-α (B), and IFN-γ (C); (D to F) frequencies of CD3^+^/CD8^+^ cells in BALB/c mice producing IL-2 (D), TNF-α (E), and IFN-γ (F). MVA, group boosted with MVA-*Pv*CelTOS; Protein, group boosted with *Pv*CelTOS protein plus Matrix-M; VLP, group boosted with *Pv*CelTOS coupled to VLPs plus the Matrix-M adjuvant. Means with SEMs are represented. *P* values were determined by one-way ANOVA followed by Tukey's multiple-comparison test. *, *P* < 0.05.

*Pv*CelTOS-specific cellular immune responses were quantified by flow cytometry after intracellular cytokine staining (ICS) of PBMCs (Fig. 2 and 3). To this end, blood samples were collected 7 days after boosting, and PBMCs were isolated and stimulated using pools of peptides whose sequences spanned the whole *Pv*CelTOS protein sequence.

In CD-1 mice, no significant differences in the levels of interleukin-2 (IL-2), tumor necrosis factor alpha (TNF-α), and IFN-γ production in CD3^+^/CD8^+^ cells were observed between nonstimulated and stimulated cells either after Ad-protein immunization or after Ad-VLP immunization (Fig. 2A to C). Peptide stimulation of cells collected after Ad-MVA prime-boost, however, induced significantly higher TNF-α levels than no stimulation of cells, with the mean values being 2.93% ± 0.72% (SD) and 0.98% ± 0.42% (SD), respectively (*P* < 0.05); likewise, for IFN-γ, the mean levels were 3.46% ± 0.699% (SD) and 1.36% ± 0.52% (SD) for stimulated and nonstimulated cells, respectively (*P* < 0.05) (Fig. 2B and C). However, the total anti-*Pv*CelTOS cellular responses in CD-1 mice obtained using this regimen were low when the background values for nonstimulated cells were subtracted from the values for stimulated cells, resulting in values of 1.9% for TNF-α and 2.1% for IFN-γ, and only the value of the latter was significantly higher than that for the naive controls (*P* < 0.0001).

In contrast to the findings obtained with CD-1 mice, all immunization regimens in BALB/c mice produced substantially higher levels of TNF-α- and IFN-γ-producing CD3^+^/CD8^+^ cells following stimulation with *Pv*CelTOS peptide pools (Fig. 2D to F). The percentage of TNF-α-positive cells after stimulation significantly increased after boosting with MVA, with means of 10.78% ± 4.16% (SD) for stimulated cells and 0.468% ± 0.314% (SD) for nonstimulated cells (*P* < 0.001); protein boosting gave mean values of 9.58% ± 4.76% (SD) for stimulated cells and 0.35% ± 0.17% (SD) for unstimulated cells (*P* < 0.001), and VLP boosting gave mean values of 11.42% ± 3.55% (SD) for stimulated cells and 1.02% ± 1.45% (SD) for unstimulated cells (*P* < 0.001). Subtraction of the background values for nonstimulated cells from the values for the stimulated cells gave mean frequencies of TNF-α-producing cells of 10.3% with MVA boosting, 9.23% with protein boosting, and 10.4% with VLP boosting (Fig. 2E). Likewise, the frequency of IFN-γ-positive cells was also high after boosting with MVA (12.82% ± 4.50% [SD] and 1.35% ± 0.40% [SD] for stimulated and unstimulated cells, respectively [*P* < 0.001]), protein (11.18% ± 5.55% [SD] and 1.13% ± 0.28% [SD] for stimulated and unstimulated cells, respectively [*P* < 0.001]), and VLPs (12.99% ± 4.33% [SD] and 1.81% ± 1.54% [SD] for stimulated and unstimulated cells, respectively [*P* < 0.001]) (Fig. 2F). Subtraction of the background values for nonstimulated cells from those for stimulated cells gave mean frequencies of IFN-γ-producing cells of 11.5% with MVA boosting, 10.1% with protein boosting, and 11.2% with VLP boosting (Fig. 2F). No significant difference in the levels of IL-2 production in CD3^+^/CD8^+^ cells was observed between nonstimulated and stimulated cells for any immunization regimen.

The induction of *Pv*CelTOS-specific CD4^+^ T-cell responses was also assessed, but no significant differences in the levels of IL-2, TNF-α, and IFN-γ production by CD3^+^/CD4^+^ cells were observed between nonstimulated and stimulated cells in either CD-1 or BALB/c mice (Fig. 3).

### Protective efficacy induced by prime-boost vaccination regimens.

To determine the protective efficacy of these four vaccination regimens, we developed a rodent challenge model, which consisted of a chimeric P. berghei parasite line [*Pb*ANKA-*Pv*CelTOS(r)_*Pb*CelTOS_ (*Pb-Pv*CelTOS)] in which the P. berghei celtos coding sequence (CDS) was replaced with the P. vivax celtos CDS (Pvceltos) (see Fig. S1 in the supplemental material). The parasites with this phenotype displayed normal growth properties and normal levels of protein expression. Parasite development and fitness are described in the supplemental material (Fig. S1).

**FIG 4:**
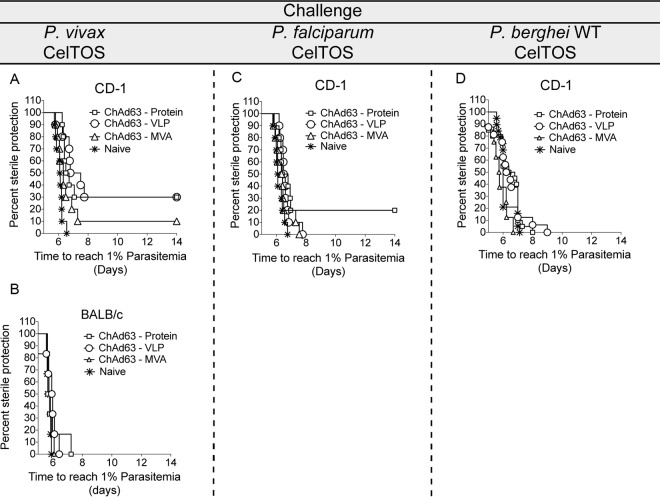
Protective efficacy in BALB/c and CD-1 mice immunized with the different vaccine platforms and challenged with chimeric and wild-type P. berghei sporozoites. Three groups of 6 BALB/c mice each and nine groups of 10 CD-1 mice each were primed with the viral vector ChAd63 (Ad) expressing *Pv*CelTOS (ChAd63-*Pv*CelTOS). The groups were subsequently boosted with (i) the MVA viral vector expressing *Pv*CelTOS (MVA-*Pv*CelTOS), (ii) the *Pv*CelTOS protein expressed in eukaryotic HEK293T cells (*Pv*CelTOS), and (iii) the *Pv*CelTOS protein conjugated to bacteriophage Qβ VLPs (VLP-*Pv*CelTOS) (see the flowchart in Fig. 1A). All mice were challenged 66 days after the boost either with wild-type (WT) P. berghei sporozoites or with chimeric sporozoites expressing *Pv*CelTOS or *Pf*CelTOS (29). (A) Protective efficacy after challenge of immunized CD-1 mice with 1,000 sporozoites of the chimeric parasite line expressing P. vivax CelTOS. Protective efficacy was significantly higher for mice immunized with Ad-protein (*P* = 0.0002) and Ad-VLPs (*P* = 0.0001) than for the naive group (log-rank Mantel-Cox test for comparison of survival curves). (B) Protective efficacy after challenge of immunized BALB/c mice with 1,000 sporozoites of the chimeric parasite line expressing P. vivax CelTOS. (C) Protective efficacy after challenge of immunized CD-1 mice with 1,000 sporozoites of the chimeric parasite line expressing P. falciparum CelTOS (29). Protective efficacy was significantly higher for mice immunized with Ad-protein (*P* = 0.0488) than for the naive group (log-rank Mantel-Cox test for comparison of survival curves). (D) Protective efficacy after challenge of immunized CD-1 mice with 1,000 sporozoites of wild-type P. berghei.

To examine the impact of the different vaccination protocols on protective immunity, the same schedule of prime-boost immunization described above was performed on BALB/c and CD-1 mice (*n* = 6 mice per group for BALB/c mice and *n* = 10 mice per group for CD-1 mice). These mice were challenged 10 days after the boost by the intravenous injection of 1,000 sporozoites either from the newly generated chimeric *Pb-Pv*CelTOS line or from wild-type P. berghei. In addition, a third set of immunized mice was challenged with P. berghei sporozoites expressing P. falciparum CelTOS (Fig. 4A to D) (29, 30). Protective efficacy was determined by measuring the prepatent period after sporozoite challenge. The prepatent period, defined as the time to reach 1% parasitemia after challenge, was calculated using a linear regression based on the three consecutive thin blood films, as described previously (32).

A modest degree of efficacy against challenge with *Pb-Pv*CelTOS was observed in CD-1 mice after boosting with either protein or VLPs (30% sterile protection); boosting with MVA protected 10% (1/10) of mice (Fig. 4A; Table 1). The protective efficacies of the Ad-protein (*P* = 0.0002) and Ad-VLP (*P* = 0.0001) regimens were significantly higher than the protective efficacy of no treatment, determined by comparison of the survival of the mice receiving these treatments with the survival of naive mice using a log-rank Mantel-Cox test to compare the survival curves. In BALB/c mice, no vaccination regimen conferred any protective efficacy against challenge with *Pb-Pv*CelTOS sporozoites, even though it induced anti-*Pv*CelTOS antibodies and *Pv*CelTOS-specific CD8^+^ T cell responses (Fig. 4B). Confirmation of the lack of efficacy was made in an additional experiment by using an increased number of mice (Fig. S2). Following a heterologous challenge with chimeric P. berghei sporozoites expressing P. falciparum CelTOS, 20% of the CD-1 mice in the protein boost group had complete protective immunity to infection (Fig. 4C). Protective efficacy was significantly higher in mice in this Ad-protein group than in the group of naive mice (*P* = 0.0488 using a log-rank Mantel-Cox test to compare survival curves). When a wild-type P. berghei challenge was used, no protective immunity from any immunization regimen was observed in CD-1 mice (Fig. 4D). A meta-analysis of the efficacy of the three vaccination regimens indicated that regardless of the parasite line used for challenge, the Ad-protein vaccine induced the highest levels of protection (Table 1), and these were significantly higher than those induced using the other immunization regimens (*P* = 0.011, one-way analysis of variance [ANOVA] with a *post hoc* Tukey analysis).

**TABLE 1:** Summary of efficacy induced by the *Pv*CelTOS vaccination regimens using Ad, MVA, protein, or Qβ VLPs in mice challenged by wild-type or transgenic P. berghei sporozoites

Challenge (1,000 sporozoites)	Vaccine regimen	Mouse strain	No. of mice protected/total no. tested	% of mice with sterile protection	Survival duration (days)			
					Median	Range	Mean ± SD	SE
*Pb-Pv*CelTOS, transgenic	ChAd63-protein	CD-1	3/10	30	6.61	6.25–14.0	8.77 ± 3.6	1.14
	ChAd63-VLPs	CD-1	3/10	30	7.15	5.80–14.0	8.96 ± 3.5	1.11
	ChAd63-MVA	CD-1	1/10	10	6.33	5.80–14.0	7.12 ± 2.5	0.78
	None^*a*^	CD-1	0/10	0	6.105	5.72–6.56	6.09 ± 0.2	0.07
*Pb-Pf*CelTOS, transgenic	ChAd63-protein	CD-1	2/10	20	6.58	5.84–14.0	7.97 ± 3.2	1.01
	ChAd63-VLPs	CD-1	0/10	0	6.535	6.18–7.76	6.63 ± 0.4	0.14
	ChAd63-MVA	CD-1	0/10	0	6.365	5.80–7.54	6.44 ± 0.6	0.18
	None	CD-1	0/10	0	6.085	5.75–6.75	6.18 ± 0.3	0.09
P. berghei wild type	ChAd63-protein	CD-1	0/20	0	6.59103	5.0–8.0	6.66 ± 0.9	0.31
	ChAd63-VLPs	CD-1	0/8	0	6.35623	5.0–9.0	6.75 ± 1.2	0.41
	ChAd63-MVA	CD-1	0/16	0	5.74167	4.99–6.69	5.81 ± 1.2	0.19
	None	CD-1	0/19	0	6	5.56–7.12	6.13 ± 0.6	0.21
*Pb-Pv*CelTOS, transgenic	ChAd63-protein	BALB/c	0/6	0	5.72106	5.63–7.24	5.99 ± 0.6	0.26
	ChAd63-VLPs	BALB/c	0/6	0	5.95633	5.56–6.43	5.95 ± 0.3	0.13
	ChAd63-MVA	BALB/c	0/6	0	5.77731	5.62–6.08	5.82 ± 0.2	0.07
	None	BALB/c	0/6	0	5.74162	3.84–5.90	5.45 ± 0.8	0.33

a The mice tested were naive.

## DISCUSSION

Efforts are under way to improve the efficacy of subunit vaccines targeting P. falciparum and P. vivax by testing new adjuvants, vaccination platforms, and antigens (33). In this study, we investigated the immunogenicity and protective efficacy of the P. vixax cell-traversal protein for ookinetes and sporozoites (*Pv*CelTOS). CelTOS is a conserved Plasmodium protein (20) that is expressed in micronemes in both ookinetes and sporozoites and plays a role in the effective traversal by ookinetes of cells in the mosquito midgut wall and in sporozoite traversal of hepatocytes (14). As a consequence, CelTOS has a critical role in the establishment of malaria parasite infections in both the mosquito and the vertebrate host. In both rodent and primate models of malaria, it has been shown that immunization strategies targeting P. falciparum or P. berghei CelTOS can enhance protective immunity (16, 17).

Here we extended those studies to the immunogenicity and protective efficacy of the Plasmodium vivax CelTOS protein. We delivered CelTOS using four clinically relevant vaccine platforms in a prime-boost vaccine approach, using ChAd63-*Pv*CelTOS (adenovirus [Ad]) as the priming agent. We demonstrated the induction of both humoral and cellular immune responses after immunization of mice with all regimens. However, despite the induction of anti-*Pv*CelTOS antibodies and *Pv*CelTOS-specific CD8^+^ T-cell responses, the various vaccine platforms demonstrated low levels of protective efficacy against challenge with chimeric P. berghei parasites expressing the *Pv*CelTOS protein.

In this study, *Pv*CelTOS-specific antibody responses were already evident after a single prime immunization with ChAd63-*Pv*CelTOS. Antibody levels increased significantly after boosting with MVA, VLPs, and protein, with the highest titers being observed after boosting with VLPs and protein. A previous study using the P. falciparum CelTOS protein showed that protein vaccination required at least two doses of adjuvanted protein to induce detectable antibody responses (17). The immunogenicity and efficacy of protein-based vaccines can be improved by priming with a recombinant Ad (31). In our study, we observed detectable antibody following priming with Ad injected intramuscularly without adjuvant. Based on these observations, we decided to use a vaccination protocol consisting of an initial Ad prime followed by a boost with one of the three other platforms, VLPs, MVA, or protein. VLPs display antigens in a repetitive and organized structure and have been shown to induce strong B-cell responses in the absence of adjuvants by efficiently cross-linking specific receptors on B cells (34). We found that the conjugate of the CelTOS protein to bacteriophage Qβ VLPs significantly boosted CelTOS antibody levels to levels comparable to those observed after boosting with protein adjuvanted with Matrix-M. The Matrix-M adjuvant is safe for use in humans. It is an immune system-stimulating complex forming 40-nm particles derived from adjuvant-active saponins, cholesterol, and phospholipids (28). Matrix-M has been shown to enhance immune responses in humans when combined with influenza or West Nile vaccines (35, 36). In our hands, the protein in Matrix-M was as efficient at boosting antibody responses as protein conjugated to bacteriophage Qβ VLPs.

As well as inducing good antibody levels, all prime-boost protocols induced high frequencies of IFN-γ- and TNF-α-producing CD8^+^ T cells in BALB/c mice. In contrast, CD8^+^ T-cell responses were low in CD-1 mice. These observations suggest the existence of an immunodominant *Pv*CelTOS epitope for inbred BALB/c mice.

The capacity of viral vectors to induce high-magnitude antigen-specific cellular immune responses against Plasmodium antigens has been demonstrated both in animal models (32, 37) and in humans (38, 39). Cellular immunity is essential for targeting of the liver stage of the parasite's life cycle (40). A prime-boost regimen using the viral vectors ChAd63 and MVA has been, to date, the most effective at inducing high-magnitude cellular immunity in humans (39). Despite the induction of both CD8^+^ T-cell and antibody responses in BALB/c mice, these immune responses did not result in any protective efficacy against challenge with *Pb-Pv*CelTOS sporozoites with any of the vaccination regimens. Previous immunization studies have reported high levels of protective immunity in mice after immunization with both P. falciparum CelTOS and P. berghei CelTOS (16, 17); protective immunity involved strong cellular and humoral components. Humoral responses could play an important role to stop the parasite cycle within the mammalian host, as CelTOS is expressed by the sporozoite and antibodies could potentially inhibit sporozoite invasion of hepatocytes. Although it was found that the transfer of increasing doses of CelTOS-specific antibodies to recipient mice resulted in incremental protective efficacy, the CelTOS-specific antibody concentrations in immunized mice did not directly correlate with the protection status of the mice (16). This suggests that the quality and the specificity of the response rather than the quantity of the response are crucial for achieving antibody-mediated protection. This may also explain the low levels of protective immunity observed in our studies, despite good antibody levels. Studies aiming to understand the B-cell epitopes responsible for the protection conferred by CelTOS can yield important information, and prediction algorithms coupled to wet lab *in vivo* models have yielded important information to contribute to the improvement of anti-CelTOS vaccine approaches (20).

The immunization strategies tested here failed to significantly increase the levels of IFN-γ- and TNF-α-producing CD8^+^ T cells in CD-1 mice. Nevertheless, we observed a modest level of protection, with 30% of the mice being protected after boosting with VLPs or protein. This may indicate that protection is mainly mediated by antibody responses. However, it is important to mention that in our study cellular responses were assessed by quantifying only the IFN-γ, TNF-α, and IL-2 secreted upon stimulation of CD4^+^ and CD8^+^ T cells with *Pv*CelTOS-specific peptides. Thus, it remains possible that other molecules, such as the cytotoxic marker CD107a, could contribute to protection. Further experiments using T-cell depletion at the time of the challenge could provide more evidence on the role of T-cell-mediated immunity. Notwithstanding the role of other T cells in protective immunity, our studies show that the vaccine platforms used are incapable of inducing strong protective immune responses in either inbred or outbred mice. This could be due to a failure to induce specific antibodies and/or T cells that recognize critical epitopes, for example, a lack of recognition of conformational epitopes by the antibodies induced by these vaccination strategies. However, it is also possible that the B- and T-cell epitopes in P. vivax CelTOS differ from those in P. berghei and P. falciparum CelTOS, which may explain the differences in protective immunity observed between our studies and other studies using the CelTOS of P. berghei and P. falciparum. Interestingly, we observed in CD-1 mice immunized with *Pv*CelTOS modest protective immunity against challenge with sporozoites expressing P. falciparum CelTOS, suggesting that cross-species protective immune responses were induced with our vaccine platforms. Further comparative studies, using different vaccine platforms and using CelTOS from different Plasmodium species, are needed to determine the value of targeting of CelTOS in a multicomponent subunit vaccine (41). Addition of adjuvants may also be a means to enhance protection.

## MATERIALS AND METHODS

### Animals.

The age-matched 6-week-old female inbred BALB/c and outbred CD-1 (ICR) strains of mice used in this study were purchased from Harlan (UK). All animals and procedures were used in accordance with the terms of the UK Home Office Animals Act Project License. Procedures were approved by the University of Oxford Animal Care and Ethical Review Committee.

### Protein expression and purification.

The mammalian codon-optimized P. vivax celtos gene (Pvceltos; gene identifier, PVX_123510) was cloned into the pHLsec plasmid with the His tag at the 3′ end under the control of the cytomegalovirus enhancer and the chick beta-actin promoter (42). DNA constructs were produced in E. coli DH5α cells (Life Technologies) and purified using an endotoxin-free plasmid megakit (Qiagen). HEK293T cells were transiently transfected using a DNA-polyethylenimine (PEI) mix. Secreted P. vivax CelTOS (*Pv*CelTOS) protein containing a C-terminal GTK(His_6_) tag was purified using a HisTrap HP 5-ml column (GE Healthcare). The His-tagged *Pv*CelTOS protein was eluted with a 0 to 1 M imidazole gradient in phosphate-buffered saline (PBS), followed by size exclusion chromatography in 20 mM Tris-HCl, pH 8.0, 300 mM NaCl. The size and purity of the purified protein were verified with colloidal Coomassie blue for total protein staining.

### Viral vector vaccines.

The mammalian codon-optimized P. vivax celtos gene (Pvceltos; gene identifier, PVX_123510) was cloned into the chimpanzee adenovirus ChAd63, and virus was grown on HEK293T cells as described earlier (43). The final concentration of the virus stock was 8.06 × 10^9^ infectious units (IU)/ml and 5.15 × 10^11^ viral particles/ml, for a ratio of the number of viral particles/number of PFU of 1:63.9.

The same *Pv*CelTOS sequence was used to develop recombinant modified vaccinia virus Ankara (MVA) expressing the *Pv*CelTOS protein. The final concentration of the virus stock was 1.7 × 10^9^ PFU/ml.

### Coupling of the *Pv*CelTOS protein to bacteriophage Qβ VLPs.

Virus-like particles (VLPs) derived from bacteriophage Qβ were expressed in E. coli JM109 containing the expression plasmid pQ10 and purified as described previously (44). The *Pv*CelTOS protein was covalently conjugated to Qβ by a two-step procedure. First, Qβ VLPs (2 mg/ml in PBS, pH 7.2) were incubated at room temperature (RT) for 30 min in the presence of a 7.5-fold molar excess of the heterobifunctional chemical cross-linker succinimidyl-6-(β-maleimidopropionamido) hexanoate (SMPH). Unreacted SMPH cross-linker was removed by diafiltration against PBS (pH 7.2) using 100-kDa Amicon Ultra centrifugal filters (Millipore). Prior to the conjugation step, purified *Pv*CelTOS was incubated for 30 min at room temperature with a 6-fold molar excess of *N*-succinimidyl-*S*-acetylthioacetate (SATA), excess SATA cross-linker was removed by diafiltration as before using 3-kDa Amicon Ultra centrifugal filters (Millipore), and derivatized protein was then deprotected using hydroxylamine (3 h at room temperature), resulting in the addition of reactive sulfhydryl residues to the protein. Following a further diafiltration, *Pv*CelTOS-SATA was covalently linked to the derivatized Qβ by reacting equimolar amounts of *Pv*CelTOS-SATA and Qβ-SMPH for 4 h at room temperature. The conjugated VLP vaccine was analyzed by SDS-PAGE, and the intensities of the Coomassie blue-stained bands corresponding to the various components of the coupling reaction were determined by densitometry and used to calculate coupling efficiency. Qβ-VLP is composed of 180 copies of the 132-amino-acid coat protein monomer. Monomeric, derivatized Qβ migrated as a discrete 15-kDa band, while the Qβ-*Pv*CelTOS conjugate migrated at ∼36 kDa (the 15-kDa Qβ monomer plus the 21-kDa *Pv*CelTOS). Coupling efficiency was defined as the molar ratio of Qβ monomers coupled to *Pv*CelTOS (the 21-kDa band) to total Qβ monomers (the sum of the 15- and 21-kDa bands). The coupling efficiency calculated in this way is a minimum estimate of the degree of coupling, as it does not take into account Qβ monomers coupled to more than one *Pv*CelTOS molecule.

### Immunization of mice.

Prior to immunization, the animals were anesthetized using an inhalation chamber containing a mixture of gases comprising isoflurane (23.5%) and oxygen (12 liters/min). Mice were initially immunized (primed) with simian adenoviral vector 63 (ChAd63) encoding the *Pv*CelTOS gene at a dose of 1 × 10^8^ IU.

At 8 weeks after priming, mice were boosted with *Pv*CelTOS-MVA, *Pv*CelTOS-protein, or *Pv*CelTOS-VLPs. Boosting with *Pv*CelTOS-MVA was performed at a concentration of 1 × 10^6^ PFU per ml. All viral vector vaccines were administered intramuscularly in endotoxin-free PBS in both limbs. All recombinant ChAd63 and MVA viral vectors used throughout this study were generated at The Jenner Institute's vector core facility.

The *Pv*CelTOS protein dissolved in PBS with Matrix-M adjuvant was administered intramuscularly. Matrix-M (Isconova, Sweden [now Novavax, MD, USA]) was mixed and briefly vortexed with the *Pv*CelTOS protein at 15 μg per dose. The adjuvant was kindly provided by Novavax, MD, USA.

The *Pv*CelTOS protein associated with bacteriophage Qβ VLPs was administered intramuscularly as a 50-μl dose containing sterile PBS and 15 μg of protein mixed with 10 μg of Matrix-M adjuvant.

### Challenge of immunized mice.

At 14 days after the boost, mice were challenged by intravenous injection of 1,000 chimeric or wild-type P. berghei sporozoites. Efficacy was assessed by calculating the prepatent period (i.e., the time to reach 1% parasitemia), as described earlier (32).

Infection of mice, sporozoite production, and isolation of sporozoites were performed as described previously (43).

### Generation of DNA constructs and genotyping of chimeric parasites expressing *Pv*CelTOS.

To generate the chimeric parasites where the P. berghei celtos (Pbceltos) coding sequence (CDS; gene identifier, PBANKA_1432300) was replaced by the P. vivax celtos (Pvceltos) CDS (gene identifier, PVX_123510), we used a 2-step gene insertion/marker out (GIMO) transfection protocol (45, 46). In the first step, we deleted the Pbceltos CDS and replaced it with the positive-negative selectable marker to create a P. berghei celtos deletion GIMO line (*Pb*ANKA-CelTOS GIMO). To do this, we generated the pL1960 construct, which is based on the standard GIMO DNA construct pL0034 (45). This construct contains the positive-negative human dihydrofolate reductase:yeast fluorocytosine uridyl (h*dhfr*::y*fcu*) selection marker (SM) cassette and was used to insert both the Pbceltos 5′ and 3′ gene targeting regions (TRs), encompassing the full-length promoter and the transcription terminator sequences, respectively. The linear pL1960 DNA construct was introduced into *Pb*GFP-Luc_con_ parasites (line 676m1cl1) using standard methods of transfection (47). Transfected parasites were selected in mice by applying positive selection by providing pyrimethamine in the drinking water (47). Transfected parasites were cloned by limiting dilution (48), resulting in the *Pb*ANKA-CelTOS GIMO line (line 2217). Correct deletion of the Pbceltos CDS was confirmed by diagnostic PCR analysis of genomic DNA (gDNA) and Southern analysis of pulsed-field gel (PFG)-separated chromosomes as described previously (47). The primers used for PCR genotyping are listed in Fig. S1 in the supplemental material.

In the second step, we replaced the positive-negative SM in the *Pb*ANKA-CelTOS GIMO genome with the Pvceltos CDS by GIMO transfection to create the P. berghei chimeric CelTOS replacement line. This was achieved by modifying the construct used in the first step (pL1960); the h*dfhr*::y*fcu* SM cassette was removed and replaced with the Pvceltos CDS sequence, generating the plasmid pL2017. The Pvceltos CDS was amplified from the DNA of a P. vivax SalI strain (Pvceltos; gene identifier, PVX_123510). The pL2017 construct was sequenced to ensure that there were no mutations in the Pvceltos CDS. The construct was linearized using ApaI and NotI restriction enzymes outside the 5′ and 3′ TRs before transfection. The construct was used to transfect parasites of a *Pb*ANKA-CelTOS GIMO line (line 2217cl1) using standard methods of GIMO transfection (45). Transfected parasites were selected in mice by applying negative selection by providing 5-fluorocytosine (5-FC) in the drinking water of the mice (46). Negative selection results in the selection of chimeric parasites where the h*dhfr*::y*fcu* SM in the celtos locus of the *Pb*ANKA-CelTOS GIMO line is replaced by the CDS of Pvceltos. Selected chimeric parasites were cloned by the method of limiting dilution. Correct integration of the constructs into the genome of chimeric parasites was analyzed by diagnostic PCR analysis on gDNA and Southern analysis of PFG-separated chromosomes as described previously (47). The primers used for PCR genotyping are listed in Fig. S1. This method creates chimeric gene replacement P. berghei parasites that lack the Pbceltos CDS but express *Pv*CelTOS [*Pb*ANKA-*Pv*CelTOS(r)_*Pb*CelTOS_; line 2320cl2] under the control of the Pbceltos regulatory sequences.

### Phenotype characterization of chimeric parasites expressing *Pv*CelTOS.

Expression of *Pv*CelTOS in the chimeric sporozoites was analyzed by immunofluorescence assay (IFA), using sera from mice immunized with the *Pv*CelTOS protein (diluted 1:100). Purified sporozoites were fixed with 4% paraformaldehyde in PBS for 20 min on ice and then washed three times with PBS and blocked with 20 μl 10% fetal calf serum (FCS) plus 1% bovine serum albumin (BSA) in PBS for 30 min at room temperature. Upon removal of the blocking medium, samples were incubated in a volume of 20 to 25 μl of the antiserum described above in 10% FCS plus 1% BSA in PBS (blocking medium) for 1 to 2 h at room temperature or overnight at 4°C. After incubation with the primary antibody, the slides were washed three times with PBS, followed by the staining with the secondary antibody Alexa Fluor 488 goat anti-mouse IgG (Life Technologies). Samples were washed three times with PBS, and nuclei were stained with Hoechst 33342 (Cell Signaling Technology) at a concentration of 2% in PBS for 10 min at room temperature, washed twice with PBS, and left to air dry, followed by addition of fluorescence mounting medium (Dako). Coverslips were mounted onto the slides, and the slides were sealed with nail polish and left to dry overnight in the dark. The parasites in both the blue and green channels were analyzed using a DMI-300B Leica fluorescence microscope, and images were processed using ImageJ software (Fig. S1).

### Peptides.

Fifteen-mer peptides overlapping by 10 amino acids spanning the whole sequence of P. vivax CelTOS were synthesized by Mimotopes Pty, Ltd., Australia. The peptides were dissolved in dimethyl sulfoxide at a concentration of 50 mg/ml and combined into a single pool for their use in intracellular cytokine staining (ICS) assays (see below) to a final peptide concentration of 5 μg/ml.

### ICS assays.

For ICS, ammonium chloride-potassium (ACK) lysis buffer-treated whole-blood PBMCs were resuspended in complete Dulbecco modified Eagle medium with 10% BSA containing 1 μl/ml GolgiPlug protein transport inhibitor and monensin and incubated for 10 h in the presence or absence of a peptide pool representing the *Pv*CelTOS antigen at individual peptide concentrations of 5 μg/ml. Phenotypic analysis of CD3^+^, CD4^+^, and CD8^+^ T cells was performed by staining PBMCs using the following antibodies: anti-mouse CD3e phycoerythrin (PE)-Cy5 (clone 145-2C11), anti-mouse CD8 peridinin chlorophyll protein-Cy5.5 (clone 53-6.7), and anti-mouse CD4 allophycocyanin (APC)-eFluor 780 (clone GK1.5); all of these antibodies were from eBioscience. For IFN-γ, TNF-α, and IL-2 cytokine staining, the following antibody clones were used: APC-conjugated rat anti-mouse IFN-γ (clone XMG1.2), Alexa Fluor 488-conjugated rat anti-mouse TNF (clone MP6-XT22), and PE-Cy7-conjugated rat anti-mouse IL-2 (clone JES6-5H4); all these clones were from BD Pharmingen. Viable cells were selected and gated using staining with stain from a Live/Dead fixable red dead cell stain kit for excitation at 488 nm (Life Technologies). Flow cytometric analyses were performed using an LSRII instrument. Data were analyzed using either FACSDiva or FlowJo software. Analysis of multifunctional CD8^+^ and CD4^+^ T-cell responses was performed using Boolean analysis in FlowJo software.

### Whole-IgG ELISA.

Enzyme-linked immunosorbent assay (ELISA) plates (F96 MaxiSorp Nunc immunoplates) were coated with 1 μg/ml *Pv*CelTOS protein (Pvceltos; gene identifier, PVX_123510) produced in HEK293T cells and purified as described above. Protein was diluted in PBS (50 μl per well), incubated at RT overnight, washed 6 times with PBS–0.05% Tween 20 (PBS-T), and blocked with PBS-T containing 10% skimmed milk (100 μl per well) for 1 h at RT. The serum was diluted 1:15,000 in PBS-T and added to duplicate wells. A standard curve was prepared with serum positive for antibodies against *Pv*CelTOS in the starting solution serially diluted 3-fold in PBS-T, and the dilutions were placed down the plate. Naive and positive serum samples were included as controls, and the samples were incubated for 2 h at RT. After incubation, the plates were washed 6 times with PBS-T. The antibody goat anti-mouse whole IgG-alkaline phosphatase conjugate (catalog number A-3562; Sigma), diluted 1:5,000 in PBS-T (50 μl per well), was added, and the plates were incubated for 1 h at RT. After incubation with anti-mouse IgG, the plates were washed 6 times with PBS-T. Then, *p*-nitrophenyl phosphate (pNPP) substrate dissolved in diethanolamine buffer (100 μl per well) was added, and the plates were incubated for 13 min at RT to allow development of the reaction. The reaction product absorbed light at 405 nm and was read on a CLARIOstar ELISA microplate reader.

### Statistical analyses.

A repeated-measure one-way ANOVA was used for comparisons among larger groups, followed by Tukey's multiple-comparison test. The Kolmogorov-Smirnov test for normality was used to determine whether the values followed a Gaussian distribution. The Mantel-Cox test was used to represent protective efficacy against challenge with chimeric P. berghei. A *P* value of <0.05 was considered statistically significant. GraphPad Prism software (San Diego, CA, USA) was used for all statistical tests.

## Supplementary Material

Supplemental material
