# Supplementary material for: Evaluation of Plasmodium vivax Cell-Traversal Protein for Ookinetes and Sporozoites as a Preerythrocytic P. vivax Vaccine
Source: Clin Vaccine Immunol. 2017 Apr 5;24(4):e00501-16. doi: 10.1128/CVI.00501-16 (PMC5382829; doi:10.1128/CVI.00501-16)
Supplement: Supplemental material [file supp_24_4_e00501-16__index.html]

Supplemental material 

# Evaluation of Plasmodium vivax Cell-Traversal Protein for Ookinetes and Sporozoites as a Preerythrocytic P. vivax Vaccine

## Supplemental material

- Supplemental file 1 -

  Fig. S1. Generation of a chimeric *P. berghei* parasite line expressing *P. vivax* CelTOS.

  PDF, 1.1M
- Supplemental file 2 -

  Fig S2. Additional assessment of the protective efficacy in BALB/c mice immunized with the Ad-MVA regimes and challenged with chimeric and wild-type *P. berghei* sporozoites.

  PDF, 29K
