# Supplementary material for: Evaluation of Plasmodium vivax Cell-Traversal Protein for Ookinetes and Sporozoites as a Preerythrocytic P. vivax Vaccine
Source: Clin Vaccine Immunol. 2017 Apr 5;24(4):e00501-16. doi: 10.1128/CVI.00501-16 (PMC5382829; doi:10.1128/CVI.00501-16)
Supplement: Supplemental material [file CVI.00501-16_zcd999095461s1.pdf]

Fig. S1

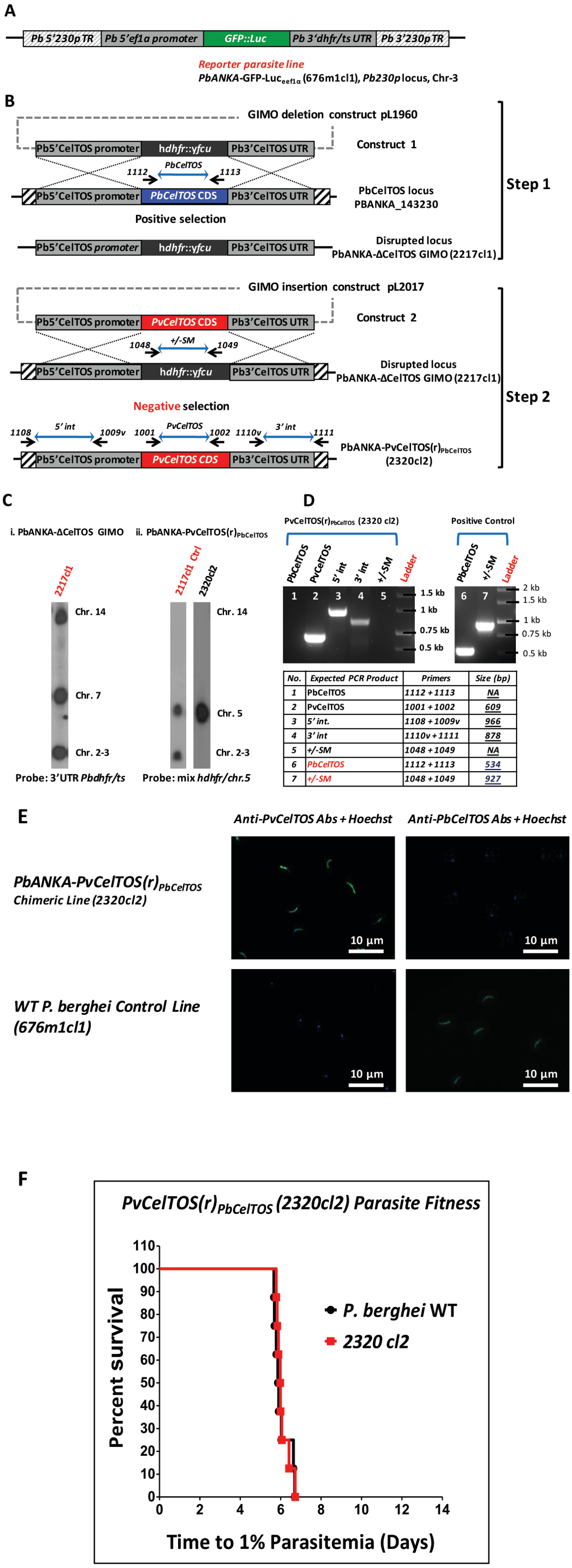

No.	Expected PCR Product	Primers	Size (bp)
1	PbCelTOS	1112 + 1113	NA
2	PvCelTOS	1001 + 1002	609
3	5' int.	1108 + 1009v	966
4	3' int	1110v + 1111	878
5	+/-SM	1048 + 1049	NA
6	PbCelTOS	1112 + 1113	534
7	+/-SM	1048 + 1049	927

Fig. S1: Generation of a chimeric *P. berghei* parasite line expressing *P. vivax* CelTOS.

(A) Schematic representation of the chimeric *Pb230p* locus of the reporter *Pb*ANKA parasite line *PbGFP-Luc*$_{Pbeef1\alpha}$ (676m1cl1) which used to generate the single replacement gene [SRG] chimeric parasite line (see **B**). This reporter line expresses a fusion protein of GFP and firefly luciferase (LUC-IAV) under the constitutive *Pbeef1a* promoter and is selectable marker (SM) free. The reporter-cassette is integrated into the neutral *230p* locus in chromosome 3.

(B) Schematic representation of the generation of the chimeric line PbANKA-PvCeLTOS(r)$_{PbCelTOS}$ (line 2320cl2) where the GIMO deletion-construct (construct 1; pL1960) is used to replace the *Pbceltos* coding sequence (CDS) with the positive/negative selectable maker (SM; h*dhfr::*y*fcu*) cassette, resulting in the generation of the *PbΔceltos* GIMO line (*Pb*ANKA-ΔCelTOS GIMO; line 2217cl1) after positive selection with pyrimethamine (step 1). Step 2: The GIMO insertion-construct (construct 2; pL2017) is used to replace the SM in the *PbΔceltos* GIMO line with the *Pvceltos* CDS after negative selection using 5-FC, resulting in the chimeric line PbANKA-PvCelTOS(r)$_{PbCelTOS}$ (line 2320cl2). Construct 2 integrates by double cross-over homologous recombination using the same targeting regions (TRs) employed in construct 1, resulting in the introduction of the *Pvceltos* CDS under the control of the *Pbceltos* gene promoter and transcriptional terminator sequences and removal of the SM. Black arrows: location and number of primers used for diagnostic PCR (see Fig. 4B).

(**C,D**) Genotype analysis of Single Replacement Gene [SRG] chimeric parasites (PbANKA-PvCelTOS(r)$_{PbCelTOS}$; line 2320cl2) and their intermediate *Pb*ΔCelTOS GIMO mother-line line (2217cl1) by Southern analysis of chromosomes (chr.) separated by pulsed-field gel electrophoresis (PFGE). **(C) Left panel: *Pb*ANKA- *ΔCelTOS GIMO:* 2217 cl1.** Hybridisation of chr. of line 2217cl1 with the 3′UTR *Pbdhfr/ts* confirms integration of construct into the *Pbceltos* gene on chr. 14. In addition, this probe hybridizes to the GFP-Luc reporter-cassette in chr. 3 and to the endogenous *Pbdhfr/ts* in Chr. 7. **Right panel: *PbANKA-PvCelTOS(r)*$_{PbCelTOS}$*: 2320cl2.*** The correct integration of the *PvCelTOS* expression construct into the GIMO locus was confirmed by showing the removal of the h*dhfr::*y*fcu* selectable marker (SM) cassette in the cloned chimeric parasite line 2320cl2. The southern blot is hybridized with a mixture of two probes: one recognizing h*dhfr* and the other is a control

probe recognizing chr. 5. As an additional control (ctrl), parasite line 2117cl1 is used with the h*dhfr::*y*fcu* SM integrated into chr-3. **(D)** Genotype analysis by diagnostic PCR analysis of the chimeric parasite line 2320cl2 confirms the correct integration of the *PvCelTOS* expression cassette. Correct integration is shown by the absence of the h*dhfr*::y*fcu* SM and the *Pbceltos* CDS, the presence of the *Pvceltos* CDS, and the correct integration of the construct into the genome at both the 5' and 3' regions (5'int and 3'int; see B for primer numbers and locations). Primers sequences used are shown in Table S1, while the expected PCR product sizes and the primer numbers are listed in the table below the PCR analysis. **(E)** Immunofluorescence analysis demonstrating *PvCelTOS* antigen expression in sporozoites of chimeric line 2320cl2. Salivary-gland sporozoites were stained with sera from mice immunized with *PvCelTOS* and *PbCelTOS* antigens (Alexa Fluor 488, green; nuclear staining with Hoechst-33342). As a control, wild-type (WT; line 676m1cl1) *P. berghei* sporozoites were stained with the same sera. Merged images of the different channels are shown for both chimeric and WT *P. berghei* sporozoites.

(F) Fitness assessment of sporozoites of the chimeric line 2320cl2. Pre-patent period in mice after injection of 1000 sporozoites of line 2320cl2 chimeric line and of the wild-type *P. berghei* parasites. The prepatent (i.e. the time to reach 1% parasitaemia) was similar in mice infected with 2320cl2 and WT sporozoites (Log-rank (Mantel-Cox) Test; P-value 0.5666.
