# Supplementary material for: Evaluation of Plasmodium vivax Cell-Traversal Protein for Ookinetes and Sporozoites as a Preerythrocytic P. vivax Vaccine
Source: Clin Vaccine Immunol. 2017 Apr 5;24(4):e00501-16. doi: 10.1128/CVI.00501-16 (PMC5382829; doi:10.1128/CVI.00501-16)
Supplement: Supplemental material [file CVI.00501-16_zcd999095461s2.pdf]

Fig. S2

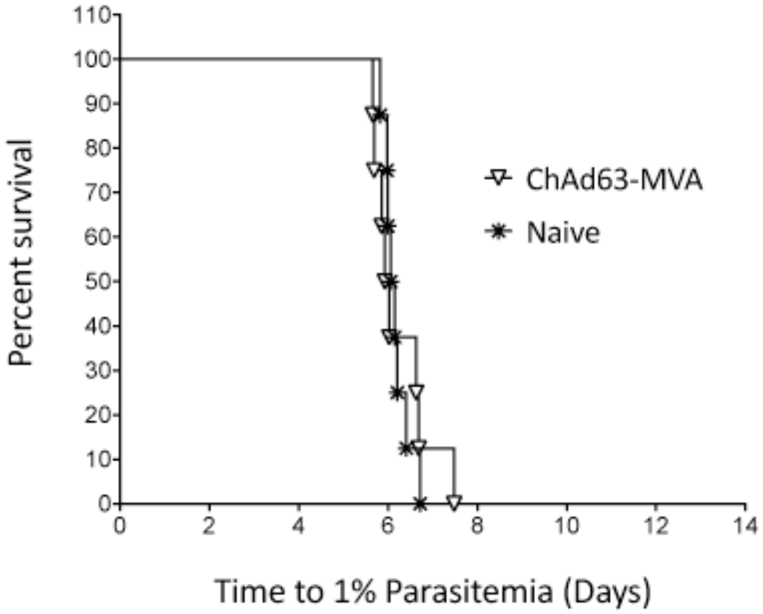

Fig S2. Additional assessment of the protective efficacy in BALB/c mice immunized with the Ad-MVA regimes and challenged with chimeric and wild type *P. berghei* sporozoites.

One group of 6 BALB/c mice was primed with the viral vector ChAd63 (Ad) vector expressing *Pv*CelTOS (ChAd63-*Pv*CelTOS) and subsequently boosted with the MVA viral-vector expressing *Pv*CelTOS (MVA-*Pv*CelTOS), All mice were challenged 66 days after the boost with 1,000 chimeric sporozoites expressing *Pv*CELTOS injected intravenously. Lack of protection in BALB/c mice by a vaccination regimen was confirmed.
